# Author Correction: Production of an immunogenic trivalent poliovirus virus-like particle vaccine candidate in yeast using controlled fermentation

**DOI:** 10.1038/s41541-026-01470-4

**Published:** 2026-05-29

**Authors:** Lee Sherry, Keith Grehan, Mohammad W. Bahar, Jessica J. Swanson, Helen Fox, Sue Matthews, Sarah Carlyle, Ling Qin, Claudine Porta, Steven Wilkinson, Suzanne Robb, Naomi Clark, John Liddell, Elizabeth E. Fry, David I. Stuart, Andrew J. Macadam, David J. Rowlands, Nicola J. Stonehouse

**Affiliations:** 1 Astbury Centre for Structural Molecular Biology, School of Molecular and Cellular Biology, Faculty of Biological Sciences, University of Leeds, Leeds, LS2 9JT UK; 2 Division of Structural Biology, University of Oxford, The Henry Wellcome Building for Genomic Medicine, Headington, Oxford, OX3 7BN UK; 3 Division of Vaccines, Medicines & Healthcare products Regulatory Agency (MHRA), Herts, EN6 3QG UK; 4 CPI, 1 Union Square, Central Park, Darlington, DL1 1GL UK; 5 Diamond Light Source, Harwell Science and Innovation Campus, Didcot, OX11 0DE UK; 6 Chinese Academy of Medical Science (CAMS) Oxford Institute (COI), University of Oxford, Oxford, OX3 7BN UK

**Keywords:** Biotechnology, Immunology, Microbiology

Correction to: *npj Vaccines* 10.1038/s41541-025-01111-2, published online 31 March 2025

In the original article, Figure 6 was incorrect and mistakenly reported immunogenicity data in which the IPV control was also adjuvanted. Figure 6 has now been updated to include the correct immunogenicity data, comparing the virus-like particle vaccine candidates to IPV in the absence of adjuvant. The corresponding figure legend has also been corrected from ‘(*, *p* value > 0.05, **, *p* value > 0.01)’ to ‘(*, *p* value > 0.05, ***, *p* value > 0.001)’ to reflect the correct statistical analysis comparing the neutralising antibody titres for the virus-like particle vaccine candidates for each serotype to IPV in the absence of adjuvant.

**Incorrect Figure 6** 
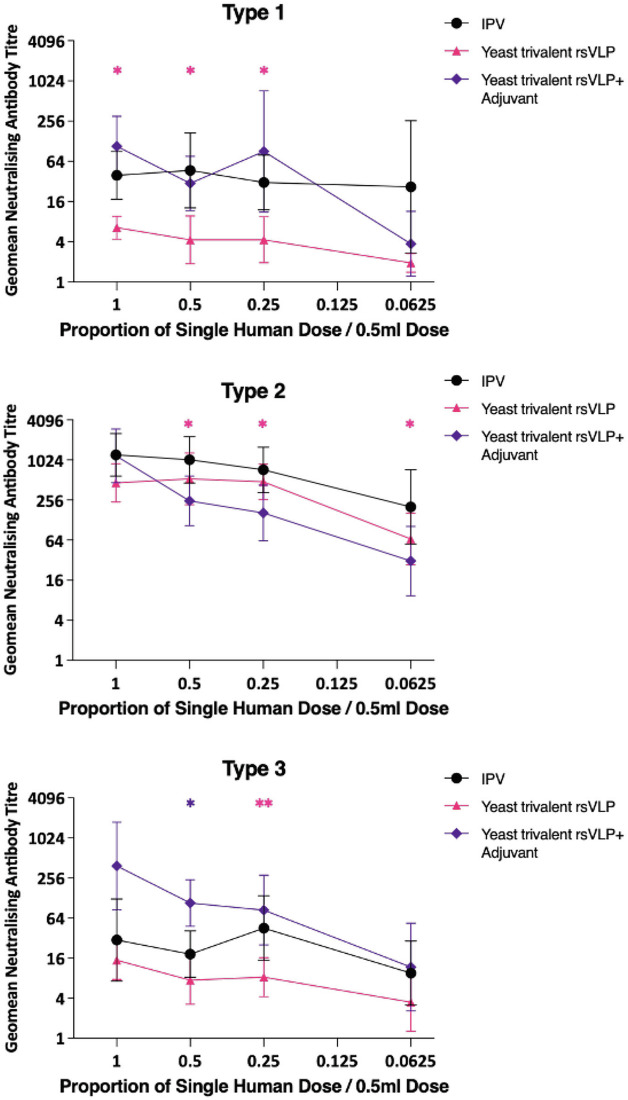


**Fig. 6 Trivalent immunogenicity of PV rsVLPs**. Dose response in neutralizing antibodies following a single immunization of Wistar rats with trivalent PV VLPs containing PV1-SC6b, PV2-SC5a and PV3-SC8 VLPs, in either the absence or presence of adjuvant. Groups of 10 rats received VLPs at various multiples of human doses and were compared to a group that had received IPV as a positive control. Sera were collected 21 dpi and neutralisation titres against the Sabin strains of PV1, PV2 or PV3 were determined. Error bars represent the Geomean Standard Deviation of the data points. Statistical analysis determined by two-tailed t test in comparison to IPV (*, *p* value > 0.05, **, *p* value > 0.01). * colour denotes the comparison group.

**Correct Figure 6** 
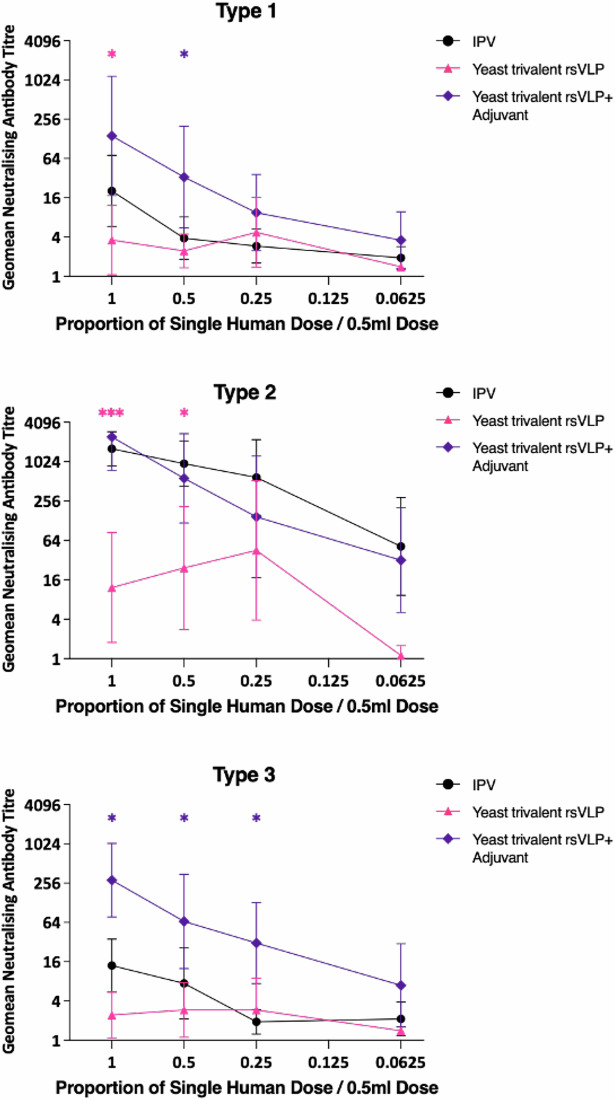


**Fig. 6 Trivalent immunogenicity of PV rsVLPs**. Dose response in neutralizing antibodies following a single immunization of Wistar rats with trivalent PV VLPs containing PV1-SC6b, PV2-SC5a and PV3-SC8 VLPs, in either the absence or presence of adjuvant. Groups of 10 rats received VLPs at various multiples of human doses and were compared to a group that had received IPV as a positive control. Sera were collected 21 dpi and neutralisation titres against the Sabin strains of PV1, PV2 or PV3 were determined. Error bars represent the Geomean Standard Deviation of the data points. Statistical analysis determined by two-tailed t test in comparison to IPV (*, *p* value > 0.05, ***, *p* value > 0.001). * colour denotes the comparison group.

