# Supplementary material for: Production of an immunogenic trivalent poliovirus virus-like particle vaccine candidate in yeast using controlled fermentation
Source: NPJ Vaccines. 2025 Mar 31;10:64. doi: 10.1038/s41541-025-01111-2 (PMC11958812; doi:10.1038/s41541-025-01111-2)
Supplement: Supplementary file 1 — Supplementary Tables [file 41541_2025_1111_MOESM1_ESM.pdf]

Supplementary Tables

Serotype	Media	Temp (℃)	Feed Conditions	VLP Yield (D Ag/100 mL culture) (n=2 +/- S.E.M)	D:C Ag Ratio
PV2-SC6b	Minimal	26	Continuous	23 (+/- 21)	N/A
		28	Continuous	5 (+/- 5)	N/A
		30	Continuous	2 (+/- 1)	N/A
PV3-SC8	Minimal	26	Continuous	7 (+/- 2)	D = C
		28	Continuous	32 (+/- 28)	D > C
		30	Continuous	4*	D = C

*** n=1**

Table S1: Minimal media Ambr250 Bioreactor production for PV2-SC6b and PV3-SC8 VLPs.

	PV2-SC6b	PV2-SC5a
	EMD-51951	EMD-51952
**Data Collection**		
Microscope	Titan Krios (eBIC)	Titan Krios (COSMIC)
Voltage (kV)	300	300
Detector	Gatan K3	Gatan K3
Recording mode	Counting	Super resolution
Nominal magnification (×)	105000	105000
Pixel size (Å) (super-resolution)	0.831	0.830 (0.415)
Defocus range (µm)	-2.3 to -0.8	-2.3 to -0.5
Dose rate (e^{-}/pixel/s)	15.16	10.80
Frames per movie	35	50
Movie exposure time (s)	1.6	3.0
Total electron dose (e^{-}/Å^2)	35.12	47.50
**Data processing**		
Movies	29172	1862
Initial particles (no.)	660747	243874
Final particles (no.)	113853	147409
Box size (pixels)	576	576
Symmetry	I1	I1
Resolution (Å)	2.4	2.1
Map sharpening *B*-factor (Å^2)	-86.1	-94.4

Table S2: Structure refinement and validation for the capsid protein (VP0, VP1, VP3)

	PV2-SC6b	PV2-SC5a
	PDB 9H93	PDB 9H94
**Model composition**		
Non-hydrogen atoms	5474	5898
Protein residues	696	724
Ligands	SPH: 1	SPH: 1
Waters		194
**Refinement**		
Resolution (Å)	2.40	2.10
Map CC[a] (Mask)	0.90	0.89
Map CC[a] (Volume)	0.86	0.86
Mean CC[a] (Ligands)	0.77	0.83
**RMS deviations**		
Bond lengths (Å)	0.004	0.003
Bond angles (°)	0.578	0.543
**Mean B-factor (Å^2)**		
Protein	19.73	13.33
Ligand	21.91	17.89
Water		15.47
**Validation**		
Molprobity[b] score (percentile)	1.02 (100th)	1.08 (100th)
Clashscore[b], all atoms (percentile)	2.03 (99th)	2.66 (98th)
Ramachandran favoured (%)	97.80	97.90
Ramachandran allowed (%)	2.20	2.10
Ramachandran outliers (%)	0.00	0.00
Rotamer favoured (outliers) (%)	95.63 (0.67)	97.91 (0.16)
Cβ deviations >0.25 Å (%)	0.00	0.00
CaBLAM outliers (%)	1.35	0.71
CA Geometry outliers (%)	0.30	0.43
**EMRinger[a,c] score**	6.00	7.50

Table S3: Structure refinement and validation for the capsid protein (VP0, VP1, VP3)

[a]Map CC and EMRinger score is given for the full particle reconstruction.

[b]Williams *et al.* (2018) Protein Sci 27:293-315.

[c]Barad *et al.* (2015) Nature Methods 12:943–946.

Supplementary Figures

A

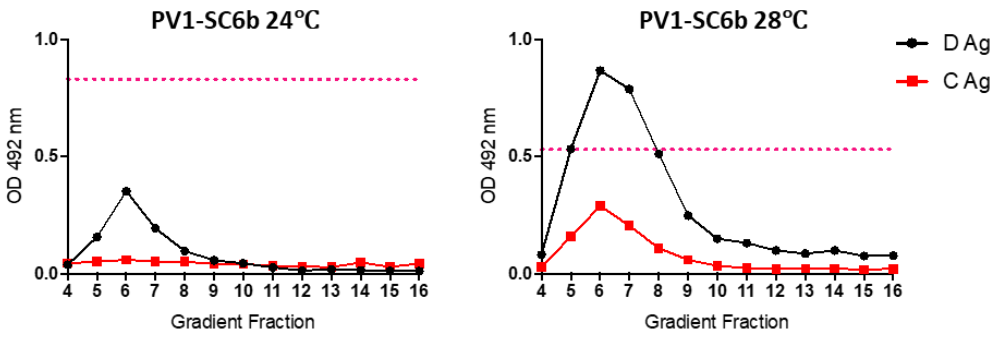

B

Serotype	Media	Temp (°C)	VLP Yield (D Ag/100 mL culture) (n=2 +/- S.E.M)	D:C Ratio
PV1-SC6b	YPD/M	24	236 (+/- 64)	D >> C
		28	2902 (+/- 630)	D > C

Supplementary Figure 1: Production of PV1-SC6b VLPs using 10 L fermentation at 24 °C vs 28 °C: A. Antigenicity of PV1 VLPs. Reactivity of gradient fractions using PV1 serotype-specific monoclonal antibodies, for D antigen or C antigen (MAb 234 and 1588, respectively) in ELISA. The pink dashed line represents the positive control, BRP, for the D antigen ELISA. OD at λ=492 nm is represented in arbitrary units. The figure is a representative example of two separate experiments for each construct. **B.** D antigen yield of PV1 VLPs following purification from 10 L bioreactor material.

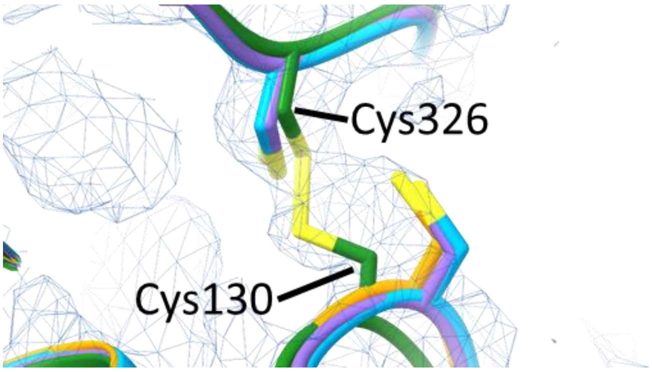

Supplementary Figure 2: Bioreactor produced PV2-SC6b disulphide bond between Cys130 – Cys326 of the capsid protomer subunit VP0. Close up view of the Cys130 and Cys326 residues in subunit VP0 of PV2-SC6b (equivalent to Cys61 and Cys257 in the mature VP2 sequence numbering), shown as sticks and coloured green. The electron potential map between Cys130-Cys326 supports the formation of a disulphide bond between the amino acid residues. Map is shown as a wire mesh at a threshold of 1.5 σ. The structures of related PV2 VLPs are shown superposed in orange (PV2-SC5a), purple (PV2-SC6b from mammalian cell expression) and cyan (PV2-SC6b from insect cell expression) (Ref. 39)
